# Chemically Laminated 2D Bis(terpyridine)metal Polymer Films: Formation Mechanism at the Liquid–Liquid Interface and Redox Rectification

**DOI:** 10.1002/chem.202201316

**Published:** 2022-07-04

**Authors:** Joe Komeda, Kenji Takada, Hiroaki Maeda, Naoya Fukui, Takuya Tsuji, Hiroshi Nishihara

**Affiliations:** ^1^ Research Institute for Science and Technology Tokyo University of Science 2641 Yamazaki Noda Chiba 278-8510 Japan; ^2^ Department of Chemistry School of Science The University of Tokyo 7-3-1, Hongo, Bunkyo-ku Tokyo 113-0033 Japan

**Keywords:** 2D materials, electron transfer, heterolayers, metal complexes, polymers

## Abstract

Recent studies on molecular 2D materials with high tunability of structure and function have focused mostly on the discovery of new precursors. Here, we demonstrate a facile one‐pot synthesis of laminated 2D coordination polymer films comprising bis(terpyridine)iron and cobalt at a water/dichloromethane interface. Cross‐sectional elemental mapping unveiled the stratum‐like structure of the film and revealed that the second layer grows to the dichloromethane side below the first layer. Cyclic voltammetry clarified that the bottom layer mediates charge transfer between the top layer and the substrate in a narrow potential region of mixed‐valence states. Furthermore, the bilayer film sandwiched by electrodes in a dry condition shows stable rectification character, and the barrier voltage corresponds to the redox potential difference between the two layers. This study introduces a new strategy for polymer design to explore the materials science of molecular 2D materials.

## Graphical abstract

**Redox rectifiers**: Heterolaminated 2D coordination polymer films were synthesized at a liquid‐liquid interface, and the mechanism of film growth was revealed. The bilayer films show unique redox behavior based on the electron transfer mediation in an electrolyte solution and stable rectification characteristics when the film is sandwiched by electrodes in a dry state.

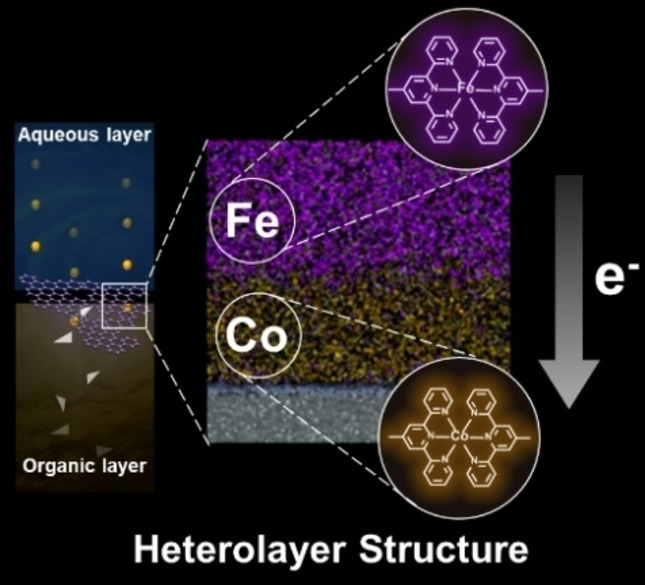

## Introduction

Two‐dimensional materials have attracted substantial attention in recent decades. Although current targets of interest are mainly inorganic materials such as graphene and transition metal dichalcogenides,[^1^, ^2^] molecular 2D materials have increased their presence in material science. They offer a wide variety of chemical structures that can be controlled by simple chemical modification of the reactants, leading to fine tuning of physical and chemical properties. Various molecular 2D materials are currently being reported including both coordination bond‐based materials (coordination nanosheets; CONASHs)[^3^, ^4^, ^5^, ^6^] and covalent bond‐based organic materials.[^7^, ^8^]

In inorganic 2D materials, vertically overlaid, so‐called van der Waals heterostructures have been developed. Heterolayer structures are common in electronic devices such as field‐effect transistors, solar cells, and light‐emitting diodes.[^9^, ^10^, ^11^, ^12^, ^13^] Therefore, it is important to establish efficient and easy synthetic methods for novel molecular 2D material heterolayers.

**Figure 1:**
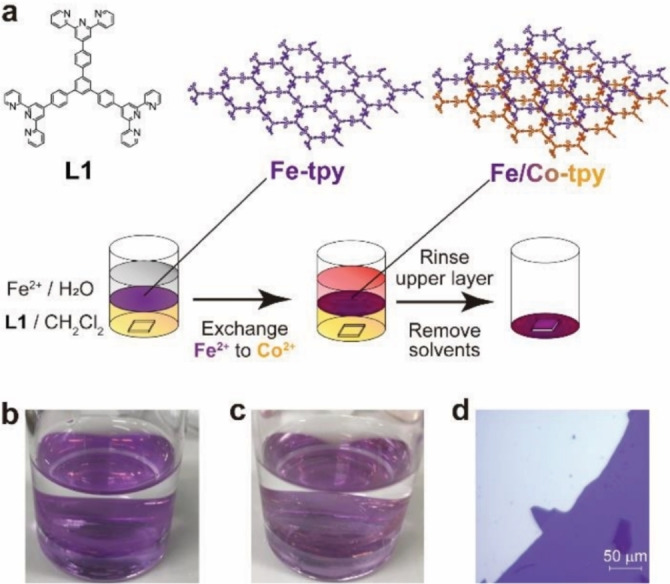
a) Schematic illustration of the sequential interfacial reaction. Photographs of b) Fe‐tpy and c) Fe/Co‐tpy. d) Optical microscopy image of Fe/Co‐tpy.

We previously reported the synthesis and electrochromism of bis(terpyridine)metal CONASHs, Fe‐tpy and Co‐tpy, by using interfacial coordination reactions.[^14^, ^15^] As bis(terpyridine)iron(II) and bis(terpyridine)cobalt(II) motifs have small dissociation reaction rate constants,^[16]^ they are suitable for stepwise construction of heterolayer structures with insignificant transmetalation.[^17^, ^18^] In the present work, we developed a sequential liquid–liquid interfacial coordination reaction method to realize structurally designable chemically laminated heterolayers (Figure 1, below). Synthesis of bis(terpyridine)metal CONASH heterolayers was carried out by allowing tris(terpyridine) ligand **L1** in dichloromethane to react sequentially with Fe^2+^ and Co^2+^ in an aqueous phase, in which the bis(terpyridine)metal polymer film with the second metal ion deposited at the surface of the film composed of the first metal ion. One of our interests in this study was to clarify the direction of coordination polymer growth, whether upward or downward. The vertical heterojunction was fully characterized by using various spectrochemical techniques and the growth direction toward the organic layer was directly visualized by using scanning transmission electron microscopy (STEM‐EDS) mapping of the heterolaminated films in cross‐section. Cyclic voltammetry and current‐voltage characteristics of the heterolaminated films were conducted to analyze electronic functions of the heterojunction of redox polymers.

## Results and Discussion

### Preparation and characterization

Preparation of bis(terpyridine)iron(II)/cobalt(II) CONASH, Fe/Co‐tpy, was performed as follows (Figure 1 and Figure S1 in the Supporting Information). First, over a dichloromethane solution of tris(terpyridine) ligand **L1**, an aqueous solution of Fe(BF_4_)_2_ was laid to fabricate a bis(terpyridine)iron(II) CONASH film (Fe‐tpy) at the interface.^[14]^ After a day, Fe‐tpy emerged as a thin purple film over the entire interface (Figure 1b). The aqueous solution was replaced with pure water. Then an aqueous solution of CoCl_2_ was added to the aqueous phase. After five days, bis(terpyridine)iron(II)/cobalt(II) CONASH (Fe/Co‐tpy) was obtained as a slightly orange‐colored purple film (Figure 1c). The as‐prepared Fe/Co‐tpy film was transferred to a substrate at the bottom of the reaction container by removing all solvents (Figure 1d). This transfer method maintains a vertical orientation, allowing the bottom surface to adhere to a variety of substrates such as Si wafers and F‐doped tin oxide (FTO)‐glass. A bis(terpyridine)cobalt(II)/iron(II) CONASH film (Co/Fe‐tpy) was prepared in the same fashion as Fe/Co‐tpy except that an aqueous solution of Co(BF_4_)_2_ was added first, and was then replaced with an aqueous solution of FeCl_2_ (Figure S2).

**Figure 2:**
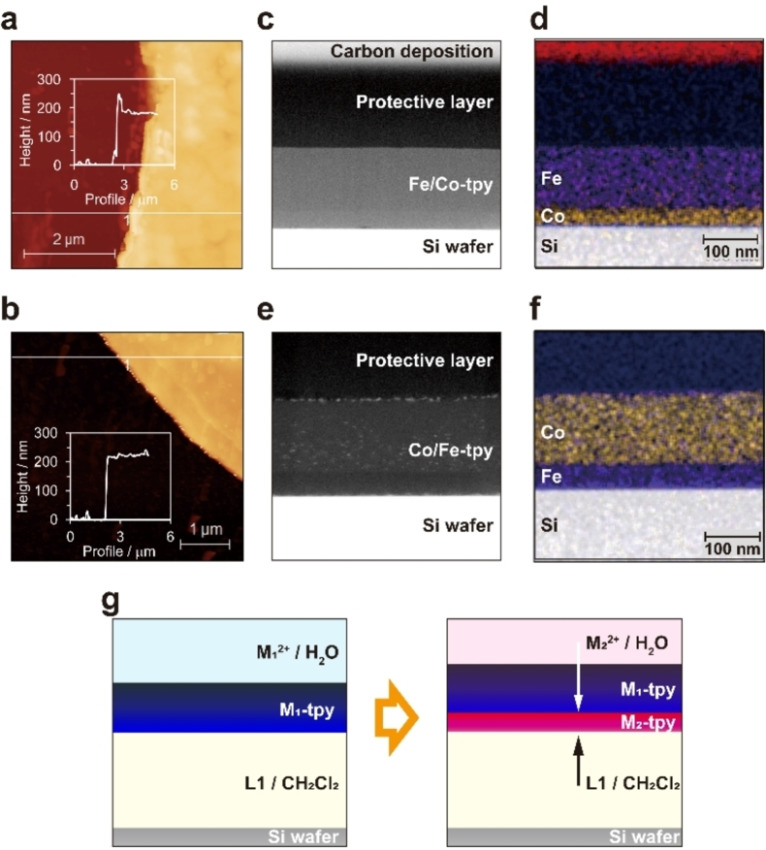
AFM image and height profile of a) Fe‐tpy and b) Fe/Co‐tpy. c) STEM image of Fe/Co‐tpy. d) STEM‐EDS elemental mapping of Fe/Co‐tpy. e) STEM image of Co/Fe‐tpy. f) STEM‐EDS elemental mapping of Co/Fe‐tpy. g) Schematic illustration of the proposed growth mechanism of the polymer.

The IR and UV‐vis spectra of Fe/Co‐tpy are similar to the sum of the corresponding Fe‐tpy and Co‐tpy spectra (Figures S3 and S4).^[14]^ Therefore, it was confirmed that Fe/Co‐tpy is composed of both [Fe(tpy)_2_]^2+^ and [Co(tpy)_2_]^2+^ moieties. Atomic force microscopy (AFM) images of Fe‐tpy and Fe/Co‐tpy (Figure 2a,b) confirm the flat surfaces of both films, and thicknesses of 180 nm for Fe‐tpy and 215 nm for Fe/Co‐tpy. The difference of 35 nm corresponds to the thickness of Co‐tpy grown in the second‐step reaction. Time‐dependence of atomic ratio in heterometallic film was observed using Fe/Co‐tpy, showing gradual growth of Co‐tpy layer (Figure S5). This result indicates that we can control the thickness of the second layer up to 60 nm. In addition, a scanning electron microscopy (SEM) image discloses flat, smooth morphology of Fe/Co‐tpy over a wide range (30 μm) and elemental analysis using SEM‐energy dispersive X‐ray spectroscopy (EDS) indicates that Fe/Co‐tpy consists of N, C, F, Cl, Fe, and Co, which are uniformly distributed throughout the film (Figure S6). Moreover, a wide‐scan X‐ray photoelectron spectroscopy (XPS) confirmed that Fe/Co‐tpy consists of N, C, F, Cl, and Fe (Figure S7), consistent with the SEM‐EDS data. The narrow scan of Co 2p core level revealed the absence of cobalt at the top surface, indicating that [Fe(tpy)_2_] is dominant at the top surface of Fe/Co‐tpy. AFM, SEM‐EDS, and XPS data of Co/Fe‐tpy indicated similar structure and composition as Fe/Co‐tpy while the way of layer stacking is reversed (Figures S11–S13).

### Formation mechanism of the heterolaminated films

Figure 2c displays a high‐angle annular dark field scanning TEM (HAADF‐STEM) image of a cross‐section of Fe/Co‐tpy. Periodic structure was not observed in the film, indicating the low crystallinity as mentioned for monometallic M‐tpy films in the previous reports.[^14^, ^19^] Inside Fe/Co‐tpy, a clear boundary in contrast parallel to the film surface was observed. In addition, STEM‐EDS mapping disclosed that cobalt is distributed on the bottom and iron is on the top (Figure 2d) with a sharp concentration‐change boundary in the line profile (Figure S8), which is consistent with the results of the spectroscopic characterization. The same metal distribution pattern was observed for Co/Fe‐tpy, where iron was distributed near the organic phase (Figures 2e, f and S14). Thus, it is concluded that the layer formed in the second step is deposited on the bottom of the first layer. This indicates that metal ions can go through the first layer from the aqueous phase to the organic phase to react with ligands at the boundary between the nanosheet and the organic phase, which would be related to the high hydrophilicity of ionic M‐tpy (Figure 2g). It is notable that this is the first clarification of the growth direction of CONASHs at a liquid‐liquid interface.

### Redox properties

**Figure 3:**
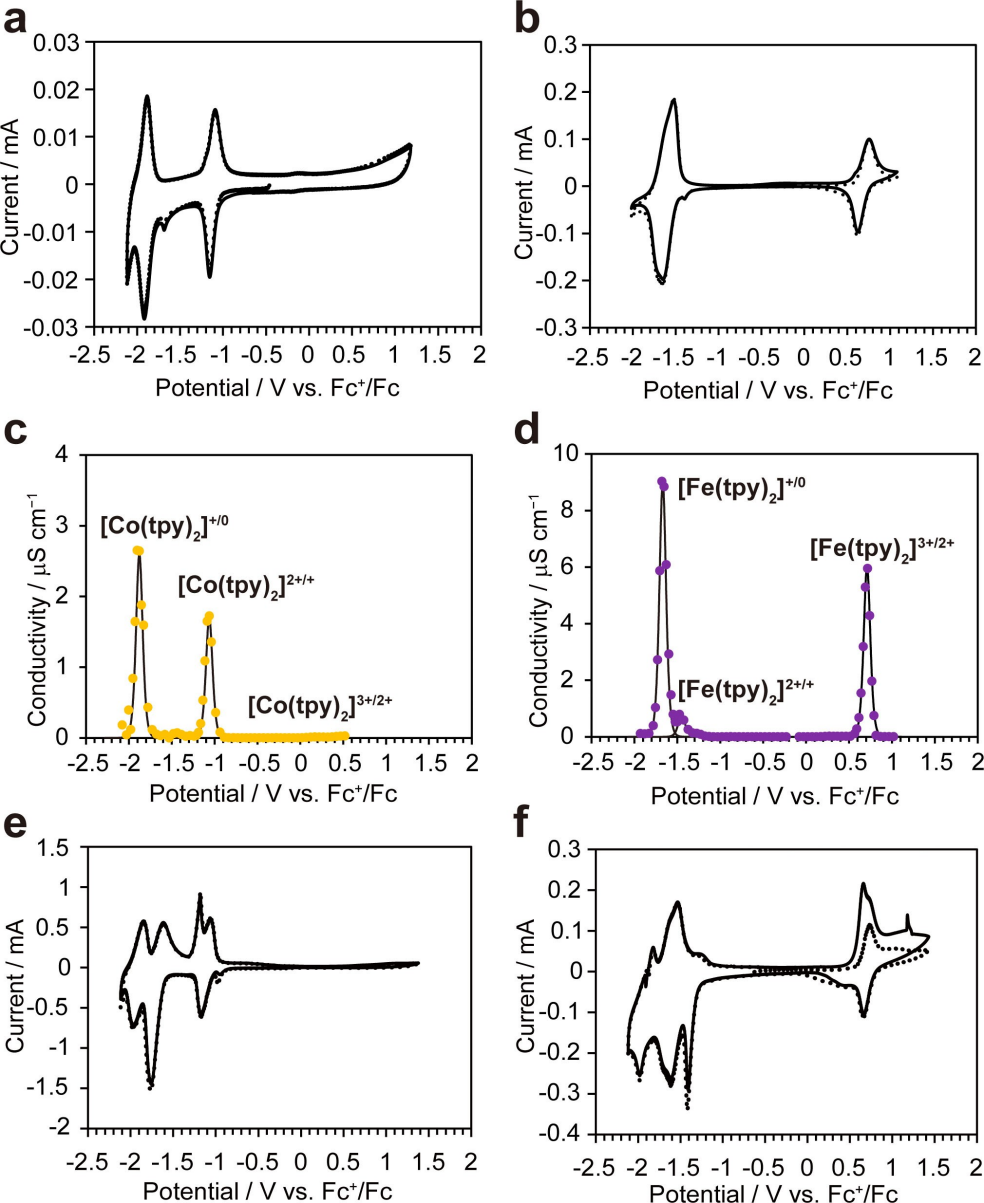
Cyclic voltammograms of monometallic polymer films a) Co‐tpy, and b) Fe‐tpy at a scan rate of 100 mV s^−1^. Potential‐dependent conductivity of c) Co‐tpy and d) Fe‐tpy. Solid lines are simulated curves based on the self‐exchange reaction model. Cyclic voltammograms of the chemically laminated films e) Fe/Co‐tpy, and f) Co/Fe‐tpy at a scan rate of 100 mV s^−1^. In cyclic the voltammograms, a dotted line represents the first cycle and a solid line the second. All electrochemical measurements were performed in 1 M *n*Bu_4_NPF_6_/CH_3_CN electrolyte.

In order to understand the redox properties of Fe/Co‐tpy and Co/Fe‐tpy (see below), we measured cyclic voltammograms of the films on FTO with a wider potential range than previously reported,^[14]^ and analyzed the potential dependency of the electrical conductivity for Co‐tpy and Fe‐tpy with the four‐electrode method using interdigitated array (IDA) electrodes in 1 M *n*Bu_4_NPF_6_ in CH_3_CN. Cyclic voltammograms of Co‐tpy show two reversible redox waves ascribed [Co(tpy)_2_]^2+/+^ at *E* ^0^’=−1.06 V vs. ferrocenium/ferrocene (Fc^+^/Fc) and [Co(tpy)_2_]^+/0^ at *E* ^0^’=−1.88 V (Figure 3a). Fe‐tpy undergoes three reversible redox reactions at *E* ^0^’=+0.72 V, −1.56 V, and −1.65 V vs. Fc^+^/Fc for [Fe(tpy)_2_]^3+/2+^, [Fe(tpy)_2_]^2+/+^, and [Fe(tpy)_2_]^+/0^, respectively (Figures 3b and S15).[^20^, ^21^] The conductivity of both films, *σ*, is below 10^−8^ S cm^−1^ in the potential range apart from their redox potentials, whereas it increases significantly in mixed‐valence states around the redox potentials (Figure 3c, d). As for Co‐tpy, the maximal σ is 1.7×10^−6^ S cm^−1^ at *E* ^0^’([Co(tpy)_2_]^2+/+^), and 2.6×10^−6^ S cm^−1^ at *E* ^0^’([Co(tpy)_2_]^+/0^) (Figure 3c). For Fe‐tpy, the maximum *σ* is 6.0×10^−6^ S cm^−1^ at *E* ^0^’([Fe(tpy)_2_]^3+/2+^), 0.8×10^−6^ S cm^−1^ at *E* ^0^’([Fe(tpy)_2_]^2+/+^), and 9.0×10^−6^ S cm^−1^ at *E* ^0^’([Fe(tpy)_2_]^+/0^; Figure 3d).

The *σ*–*E* characteristic of redox polymer films is interpreted by the electron self‐exchange reaction model.^[22]^ The electron self‐exchange rate constant, *k*_ex_ for [Fe(tpy)_2_]^3+/2+^ in Fe‐tpy is estimated at 1.4×10^3^ M^−1^ s^−1^, which is on the same order as *k*_ex_ for [Fe(tpy)_2_]^3+/2+^ in dendritic molecular wires^[23]^ on the electrode surface, 9.9×10^3^ M^−1^s^−1^ (see Supporting Section D). This is reasonable because the electron transfer process of the Fe‐tpy film involves not only fast, through‐bond electron transfer within the sheet, but also slow through‐space electron transfer between sheets.

Cyclic voltammetry of the heterolaminated Fe/Co‐tpy and Co/Fe‐tpy films showed characteristic redox behaviors that were not observed in homometallic polymer films. A cyclic voltammogram of Fe/Co‐tpy on FTO shows a reversible wave for the [Co(tpy)_2_]^2+/+^ couple (*E* ^0^’=−1.11 V), but no peaks for the [Fe(tpy)_2_]^3+/2+^ couple are observed around +0.74 V. This clearly indicates that the redox reaction of the outer Fe‐tpy layer is blocked by the inner Co‐tpy layer, which does not conduct in the [Fe(tpy)_2_]^3+/2+^ redox potential region. However, compared with the cyclic voltammogram of Co‐tpy, a remarkable difference appears in the potential range between −1 and −2 V versus Fc^+^/Fc in the cyclic voltammogram of Fe/Co‐tpy (Figure 3e). The redox waves at *E* ^0^’=−1.11 V, and −1.90 V are attributed to [Co(tpy)_2_]^2+/+^ and [Co(tpy)_2_]^+/0^, respectively, of the inner layer, whereas an additional wave at *E* ^0^’=−1.70 V is assignable to the [Fe(tpy)_2_]^2+/+^ couple of the outer layer, indicating that the inner Co‐tpy layer conducts sufficiently to mediate the redox reaction of the outer Fe‐tpy layer in the [Fe(tpy)_2_]^2+/+^ redox potential region. In addition, a charge‐state‐trapping anodic peak appears at −1.18 V, which is typical of redox bilayer films for mediating electron transfer of the outer layer by the redox‐active inner layer.^[24]^ The above discussion is consistent with the result of the potential dependence of the conductivity of Co‐tpy, as noted above (Figure 3a).

Co/Fe‐tpy showed similar behavior as Fe/Co‐tpy. In addition to three reversible redox waves of the inner Fe‐tpy layer, a remarkable charge‐state‐trapping cathodic peak for [Co(tpy)_2_]^2+/+^ appears at −1.41 V. Potential dependence of the conductivity of Fe‐tpy (Figure 3d) indicates that this phenomenon occurred by electron transfer mediation when the inner Fe‐tpy layer becomes conductive.

It should be noted that the above‐mentioned complete electrochemical redox rectification property is due to the inner film having sufficient thickness. In the two‐dimensional polymer material system, the relationship between the film thickness and the redox rectification characteristics has not been known, but in the 1D bis(terpyridine)iron and cobalt polymer system with a heterostructure, when the inner wire is short (ca. 10 nm), the redox of the outer wire is not completely irreversible due to the rapid redox hopping through the inner wire.^[25]^ But if the inner wire is long enough (ca. 10 μm), the rectification characteristics is remarkable.^[26]^ In this study, it is possible to control the inner film thickness up to 60 nm (see above), and we believe that it is possible to clarify the relationship between the film thickness and the redox rectification characteristics as the next step.

### Rectification properties

**Figure 4:**
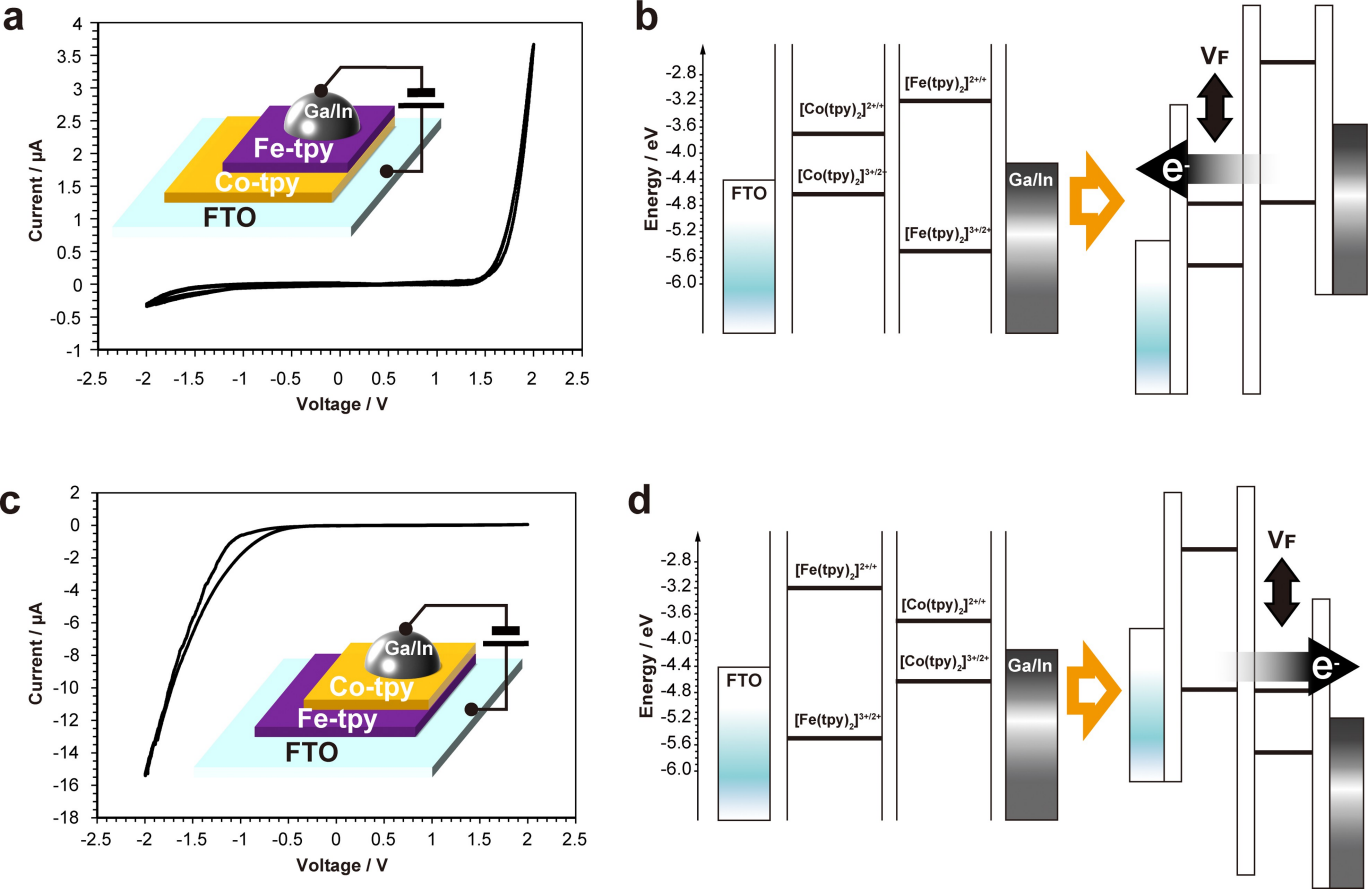
*i*–*V* curves of a) Fe/Co‐tpy and c) Co/Fe‐tpy; Insets: schematic illustrations of the experimental setup for each chemically laminated film. Rectification mechanism explained from the viewpoint of energy diagrams of b) Fe/Co‐tpy and d) Co/Fe‐tpy at zero bias (left) and applied forward bias (right).

Electrical conductivity measurements of Fe‐tpy, Co‐tpy, Fe/Co‐tpy and Co/Fe‐tpy were carried out for films on FTO by connecting the surfaces of the films to gallium‐indium (Ga/In) alloy. Fe‐tpy and Co‐tpy nanosheets showed nonlinear and symmetric *i*–*V* curves (Figure S16), which are typical behaviors for semiconducting organic materials.[^27^, ^28^] On the other hand, Fe/Co‐tpy shows clear rectification behavior, in which the current increases sharply when a voltage of 1.8 V is applied (Figure 4a). In contrast, Co/Fe‐tpy shows an opposite rectification behavior such that the negative current increases significantly at a voltage lower than −1.2 V (Figure 4c). These results suggest that the Fe‐tpy/Co‐tpy junction works as a diode with its forward current direction from Co‐tpy to Fe‐tpy.[^29^, ^30^]

This rectification behavior of the heterolayers is explained by energy diagrams based on energy levels of [Co(tpy)_2_]^2+/+^ and [Co(tpy)_2_]^3+/2+^ of Co‐tpy, [Fe(tpy)_2_]^2+/+^ and [Fe(tpy)_2_]^3+/2+^ of Fe‐tpy, and work functions of FTO^[31]^ and Ga/In alloy,^[32]^ (Figures 4a, c). For forward bias (*V*_F_), current is allowed to flow around *V*_F_=2.1 V, where electron transfer takes place from Fe‐tpy to Co‐tpy (Figure 4b). Note that an excess of 0.3 V is needed to compensate for the difference between the work functions of FTO and Ga/In, in addition to the energy level difference between [Fe(tpy)_2_]^3+/2+^ and [Co(tpy)_2_]^2+/+^. This two‐level system fully explains the experimental data. On the other hand, for reverse bias, apparently, current is allowed to flow as well if only the energy diagram is considered. However, since oxidation of [Co(tpy)_2_]^2+^ is slow, as observed in the cyclic voltammogram (Figure 3a),[^14^, ^33^] electron transfer hardly occurs. Finally, we refer to Co/Fe‐tpy, in which rectification behavior was observed as well, but the threshold voltage was reduced compared with the Fe/Co‐tpy device (Figure 4d) due to the work function difference between FTO and Ga/In. The *i*–*V* characteristics can be characterized by redox conduction theory, in which both Fe‐tpy and Co‐tpy are in the mixed valence state, [Fe(tpy)_2_]^3+/2+^ and [Co(tpy)_2_]^2+/+^, respectively. Current flows when a mixed valence state occurs at the interface by applying a voltage.

## Conclusion

The facile synthesis of heterolaminated 2D polymer films was achieved by sequential liquid–liquid interfacial coordination. The growth direction was directly visualized by STEM‐EDS mapping. Cyclic voltammetry of the heterolaminated films exhibited specific redox behaviors in which the inner layer mediates the redox reaction of the outer layer in a potential region where the mixed‐valence state of the inner layer is formed and becomes conductive. The heterolaminated films work as redox rectifiers ascribed to the potential difference at the junction of heterolayers. Our findings will give insights into chemical phenomena occurring at liquid–liquid interfaces, such as chemical reactions and mass transport. Moreover, our study will expand the structural design of molecular 2D polymer films leading to their further development for materials science.

## Experimental Section

**Materials**: All chemicals were purchased from Tokyo Chemical Industry Co., Kanto Chemical Co., Sigma–Aldrich, or FujiFilm Wako Chemical Corporation without further purification, unless otherwise stated. Water was purified with a Milli‐Q purification system (Merck). As a substrate for IR spectroscopy, AFM, SEM‐EDS, XPS, and STEM‐EDS, a Si (111) wafer (n‐type, P‐doped, 0.03–0.04 Ωcm) was purchased from Electronics and Materials Corporation. As a substrate for cyclic voltammetry, a commercially available FTO‐glass substrate was used. For UV‐vis absorption spectroscopy, a quartz plate was used. Terpyridine ligand (**L1**) was synthesized according to the literature.^[34]^ Tetra‐*n*‐butylammonium hexafluorophosphate (*n*Bu_4_NPF_6_) was purchased from Tokyo Chemical Industry Co., Ltd., and recrystallized from hot ethanol, then dried in vacuo. Ga/In alloy was prepared by keeping the mixture of gallium and indium metals in a ratio of 4 to 1 in weight at 120 °C for 1 h.

**Instruments**: Optical microscopic images were taken using an optical digital microscope (VHX‐1000, Keyence) equipped with a VH−Z75 (Keyence). The Fourier transform infrared (FTIR) spectra were obtained in attenuated total reflection method using a Nicolet iS50 FTIR (ThermoFisher Scientific). UV‐vis absorption spectra were measured using a JASCO V‐570. AFM measurement was carried out using an Agilent Technologies 5500 Scanning Probe Microscope with a silicon cantilever PPP‐NCL or NCH (Nano World) in the high amplitude mode (Tapping Mode) under an ambient condition. SEM‐EDS was conducted using a JEOL JCM‐7000 with an acceleration voltage of 15 kV. XPS were measured by using a Kratos AXIS Nova or an ULVAC PHI VersaProbe. Al Kα was used as an X‐ray source. The obtained spectra were calibrated to be C 1s=284.6 eV. A series of electrochemical measurements was conducted using ALS 650DT or ALS 750E electrochemical analyzers at room temperature (25 °C). Cyclic voltammetry and differential pulse voltammetry were measured in a 1 M *n*Bu_4_NPF_6_ solution of CH_3_CN as a supporting electrolyte solution. A Pt wire and an Ag^+^/Ag reference electrode (10 mM AgClO_4_ in 0.1 M *n*Bu_4_NClO_4_/CH_3_CN) were used as a counter electrode and a reference electrode, respectively. Simulation of the voltammograms was carried out on a program equipped in CHI ALS 750E software. In electronic conductivity measurement through vertical direction, glassy carbon (GC) electrode was attached on the top of Fe/Co‐tpy deposited on FTO‐glass substrate (Figure 4a inset). Ga/In alloy was used to confirm the contact between GC electrode and Fe/Co‐tpy. The voltage was applied to the FTO‐glass. STEM‐EDS measurement of cross‐section of Fe/Co‐tpy was measured by using a JEOL‐ARM200F thermal FE electron gun with an acceleration voltage of 200 kV. The specimen was protected by permanent marker and carbon deposition then cut off by focused ion beam (FIB) using a Fischione Instruments Model 1040 or JEOL JIB‐4600F.

**Preparation of Fe/Co‐tpy**: A 0.1 mM solution of **L1** in CH_2_Cl_2_ was prepared by dissolving 1 mg of **L1** into 10 mL of CH_2_Cl_2_. The solution was filtrated by using an Advantech disposable filter unit, then poured into vial with a diameter of 40 mm. At the bottom of the vial, substrates such as FTO‐glass and silicon wafer were laid. After that, 10 mL of pure water was added gently on the top of dichloromethane solution to form water/organic solvent interface. An aqueous solution of Fe(BF_4_)_2_ (50 mM, 10 mL, filtrated prior to use) was added gently to the water. After a day, Fe‐tpy formed on the interface as a purple film, which was collected by the method discussed below, or followed by replacement of the Fe(BF_4_)_2_ solution to pure water, then a CoCl_2_ solution (50 mM, 10 mL, filtrated prior to use). 5 days after the replacement, the obtained film became a bit more orange. The aqueous layer was replaced with pure water and pure EtOH, which was followed by removal of both aqueous and organic phases to allow Fe/Co‐tpy to cover the substrates. The substrates covered by Fe/Co‐tpy were washed with EtOH.

**Preparation of Co/Fe‐tpy**: Co/Fe‐tpy was prepared in the same fashion as Fe/Co‐tpy. Instead of Fe(BF_4_)_2_, an aqueous solution of Co(BF_4_)_2_ (50 mM, 10 mL, synthesized by anion exchange between CoCl_2_ and AgBF_4_, accompanied by by‐product AgCl, which was removed by filtration) was first added. After two days, Co‐tpy formed as an orange film, which was used for various analyses or followed by replacement of the Co(BF_4_)_2_ solution to pure water, then an aqueous FeCl_2_ solution (50 mM, 10 mL, pH 2 adjusted with HCl, filtrated prior to use). Four days after the replacement, the obtained film became a little more purple. Co/Fe‐tpy was transferred to various substrates in the same fashion as Fe/Co‐tpy.

## Conflict of interest

The authors declare no conflict of interests.

1

## Supporting information

As a service to our authors and readers, this journal provides supporting information supplied by the authors. Such materials are peer reviewed and may be re‐organized for online delivery, but are not copy‐edited or typeset. Technical support issues arising from supporting information (other than missing files) should be addressed to the authors.

Supporting Information

## Data Availability

The data that support the findings of this study are available in the Supporting Information.
