# Supplementary material for: Chemically Laminated 2D Bis(terpyridine)metal Polymer Films: Formation Mechanism at the Liquid–Liquid Interface and Redox Rectification
Source: Chemistry. 2022 Jul 4;28(44):e202201316. doi: 10.1002/chem.202201316 (PMC9400887; doi:10.1002/chem.202201316)
Supplement: Supplementary file 1 — Supporting Information [file CHEM-28-0-s001.pdf]

Chemistry–A European Journal

Supporting Information

Chemically Laminated 2D Bis(terpyridine)metal Polymer Films: Formation Mechanism at the Liquid–Liquid Interface and Redox Rectification

Joe Komeda, Kenji Takada, Hiroaki Maeda, Naoya Fukui, Takuya Tsuji, and Hiroshi Nishihara*

Table of Contents

A. Schematic illustration for the synthesis of **Fe/Co-tpy** and **Co/Fe-tpy**

B. Characterization of **Fe/Co-tpy**

C. Characterization of **Co/Fe-tpy**

D. Analysis of potential-dependent conductivity of **Fe-tpy** and **Co-tpy**

E. Electrochemical analysis of **Fe/Co-tpy**

F. Electrical conductivity measurement on **Fe/Co-tpy** and **Co/Fe-tpy**

A. Schematic illustration for the synthesis of Fe/Co-tpy and Co/Fe-tpy

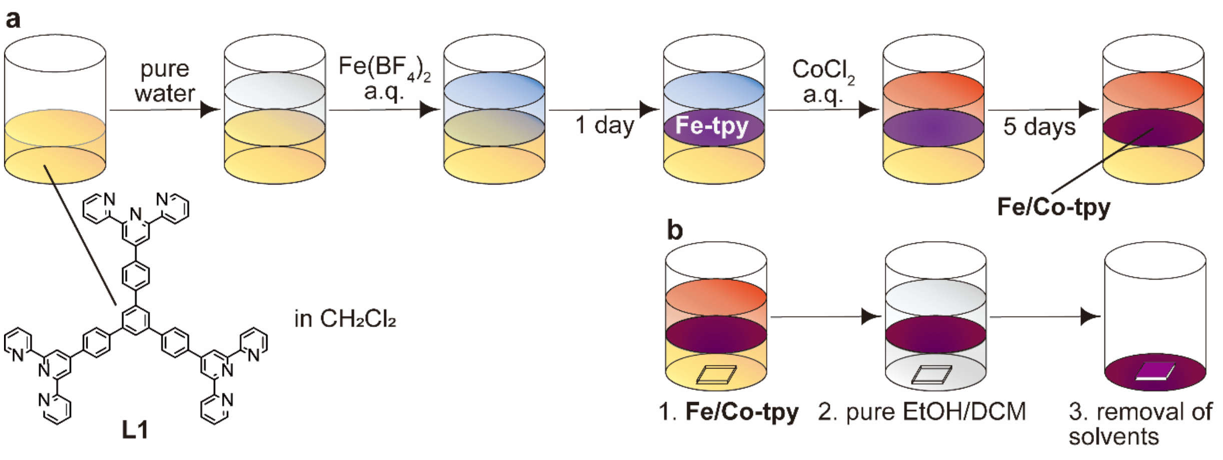

Figure S1. Detailed schematic illustration of sequential liquid/liquid interfacial reaction of **Fe/Co-tpy** (a) and transfer method (b).

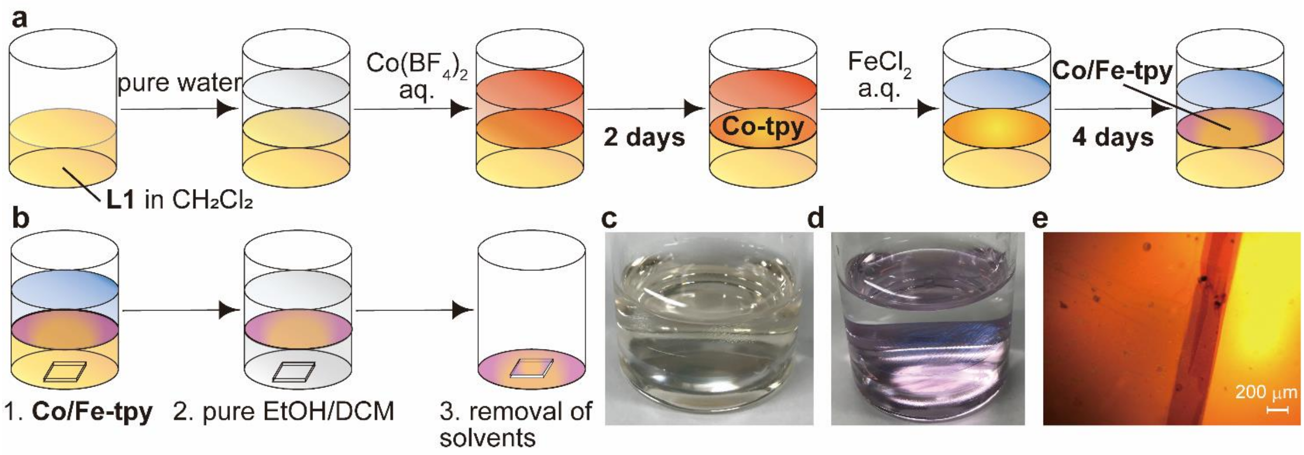

Figure S2. (a,b) Detailed schematic illustration of sequential liquid/liquid interfacial reaction (a) and transfer method (b). (c,d) Picture of (c) **Co-tpy** and (d) **Co/Fe-tpy**. (e) Optical microscopic image of **Co/Fe-tpy**.

B. Characterization of Fe/Co-tpy

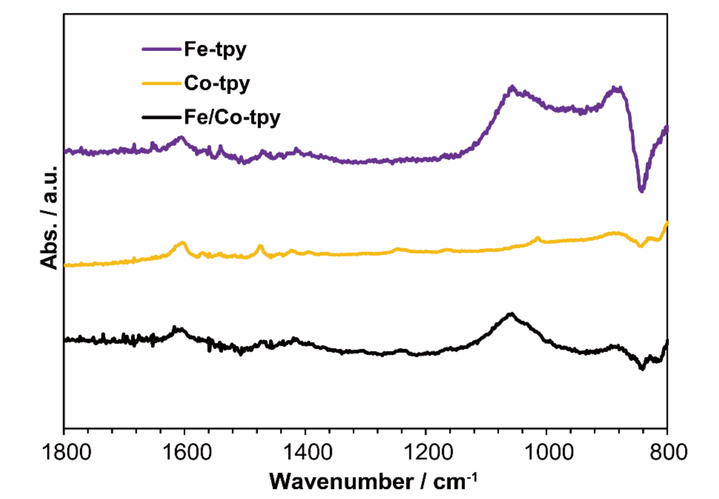

Figure S3. IR spectra of **Fe-tpy** (purple), **Co-tpy** (orange), and **Fe/Co-tpy** (black).

In the IR spectrum of **Fe/Co-tpy**, a C=C stretching at 1600 cm^{-1} was observed for **Fe-tpy**, **Co-tpy**, and **Fe/Co-tpy**, which indicates the existence of bridging terpyridine ligand. A broad peak at 1100 cm^{-1} ascribed to B-F stretching of BF_4^- was observed for **Fe-tpy** and **Fe/Co-tpy**.

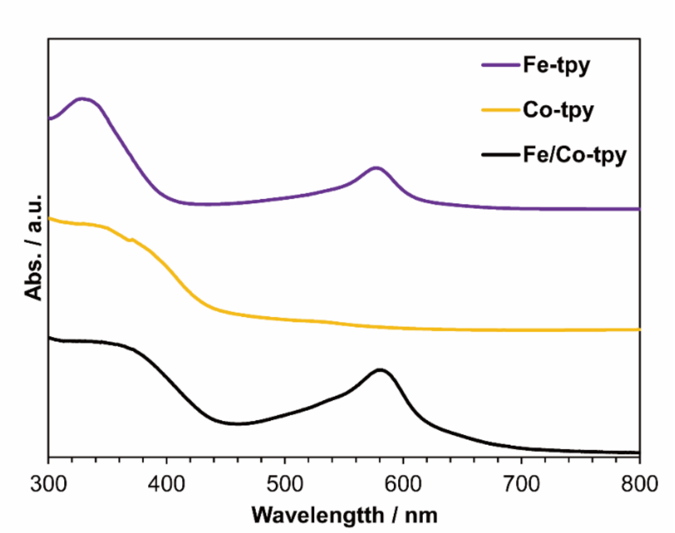

Figure S4. UV-vis absorption spectra of **Fe-tpy** (purple), **Co-tpy** (orange), and **Fe/Co-tpy** (black).

The UV-vis absorption spectrum of **Fe/Co-tpy** shows the peak at λ_{max} = 578 nm, which is attributed to metal-to-ligand charge transfer (MLCT) absorption of bis(terpyridine)iron(II) center ($[Fe(tpy)_2]^{2+}$), as well as a broad peak around 300-450 nm corresponding to $\pi-\pi^*$ transition characteristic to $[Co(tpy)_2]^{2+}$.

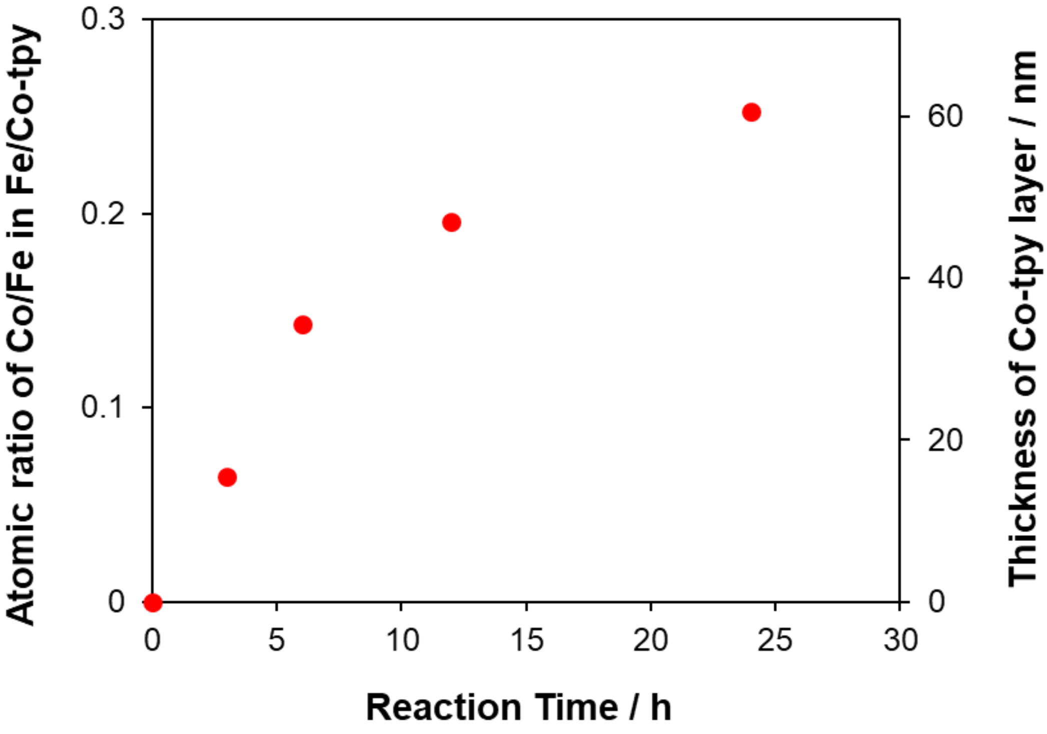

Figure S5. Time-dependent atomic ratio of Co during the growth of **Co-tpy** layer on **Fe-tpy** layer with the thickness of 240 nm. Atomic ratio was determined using EDS equipped to SEM. Thickness of **Co-tpy** was calculated by multiplying the atomic ratio to the thickness of **Fe-tpy** layer.

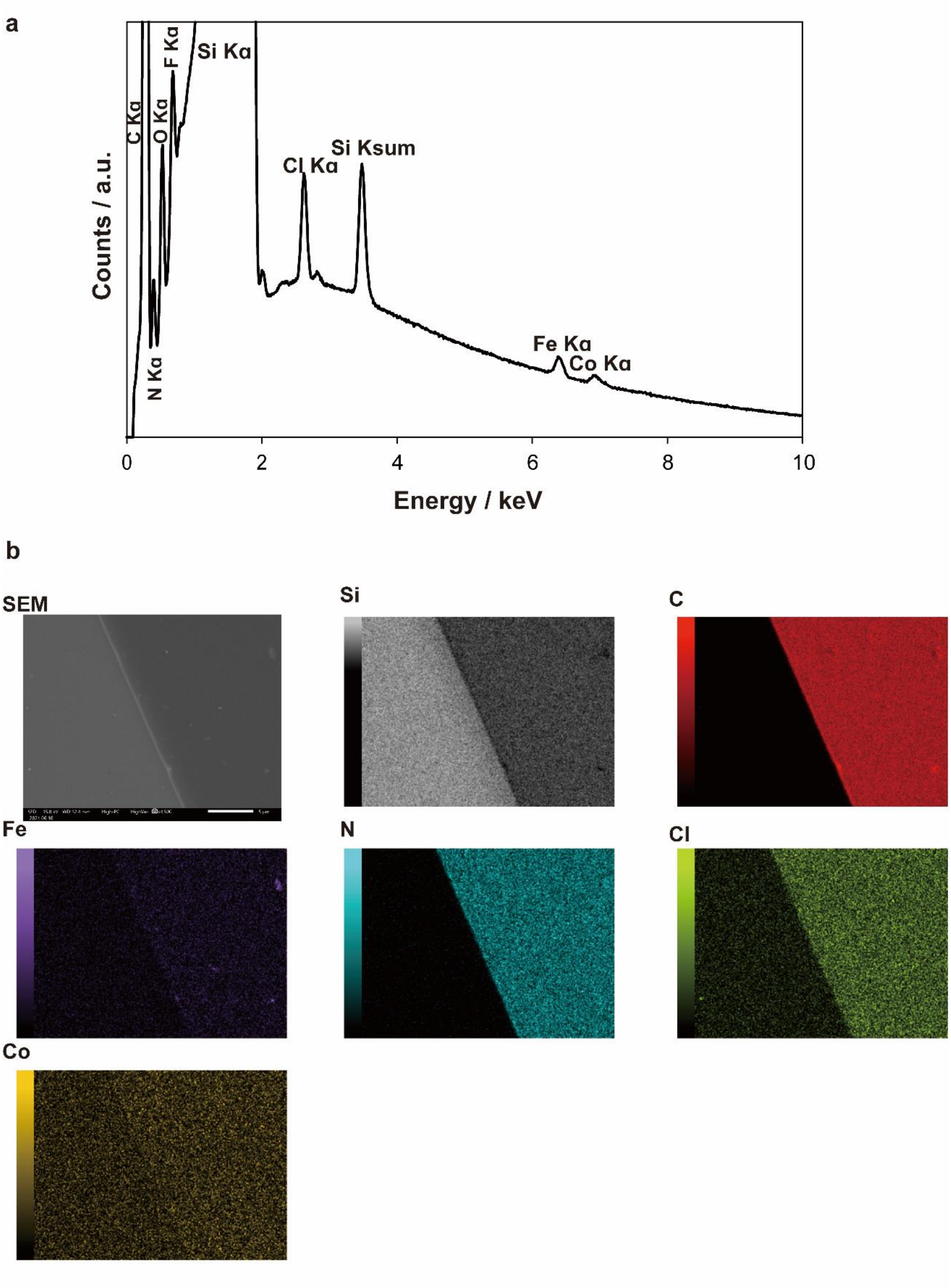

Figure S6. SEM-EDS of **Fe/Co-tpy**. (a) Spectrum. (b) SEM image and EDS mapping of each element.

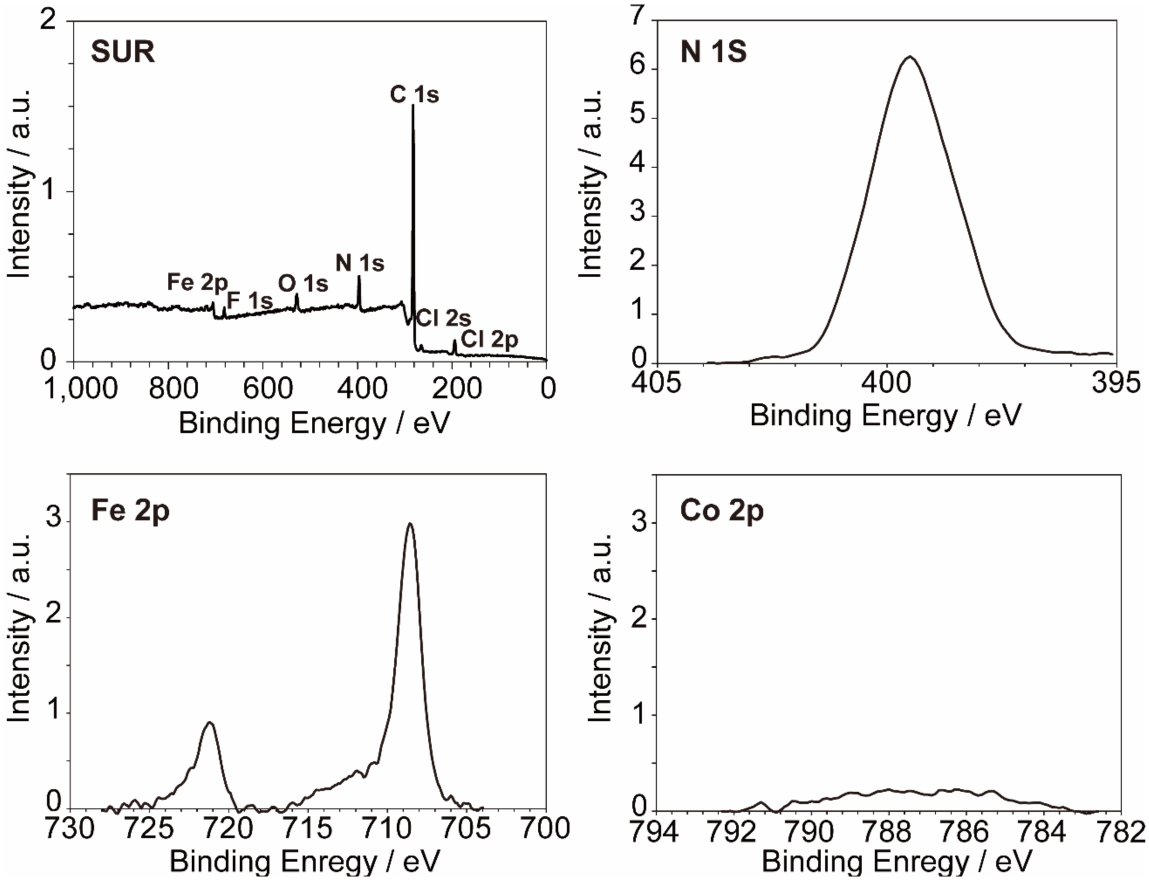

Figure S7. XPS on **Fe/Co-tpy**. Survey scan (top left), Narrow scan of N1s (top right), Fe 2p (bottom left), and Co 2p (bottom right).

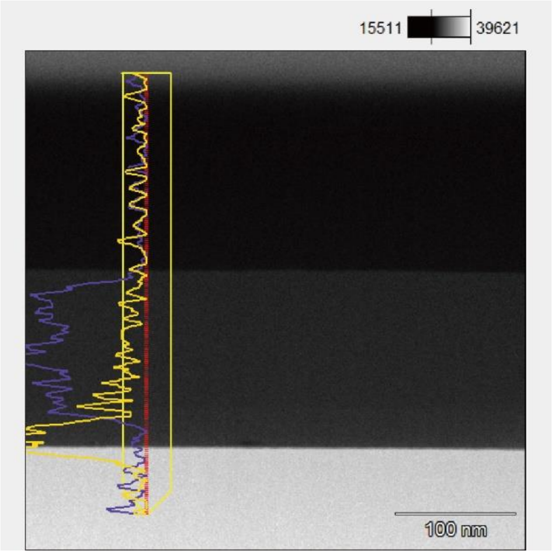

Figure S8. STEM image and EDS line profile across indicated region of **Fe/Co-tpy**. Purple: Fe, Orange: Co.

C. Characterization of Co/Fe-tpy

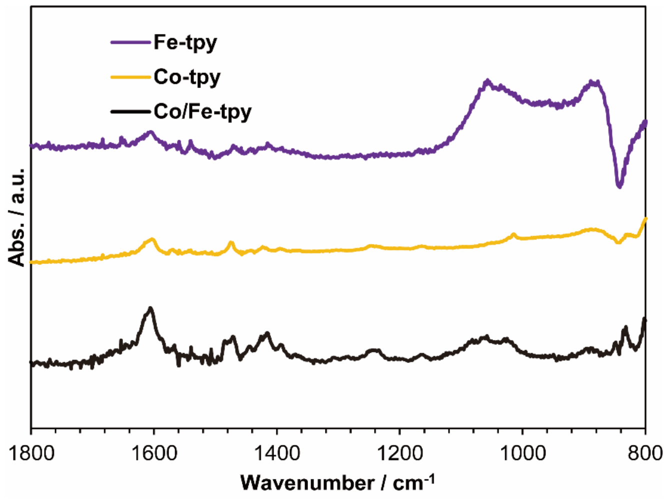

Figure S9. IR spectra of **Fe-tpy** (purple), **Co-tpy** (orange), and **Co/Fe-tpy** (black).

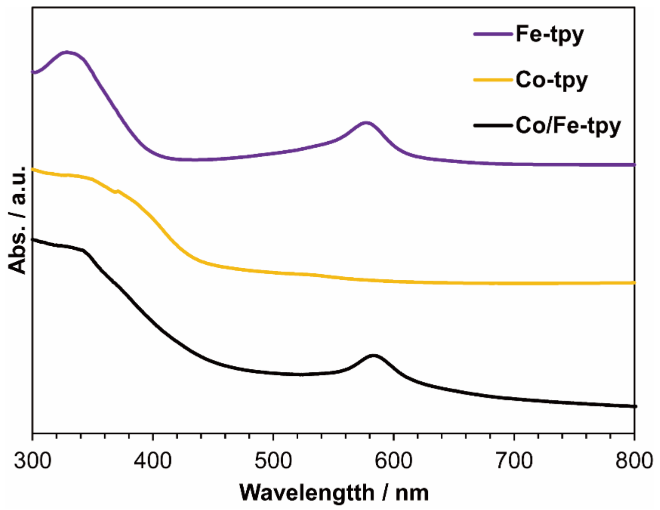

Figure S10. UV-vis absorption spectra of **Fe-tpy** (purple), **Co-tpy** (orange), and **Co/Fe-tpy** (black).

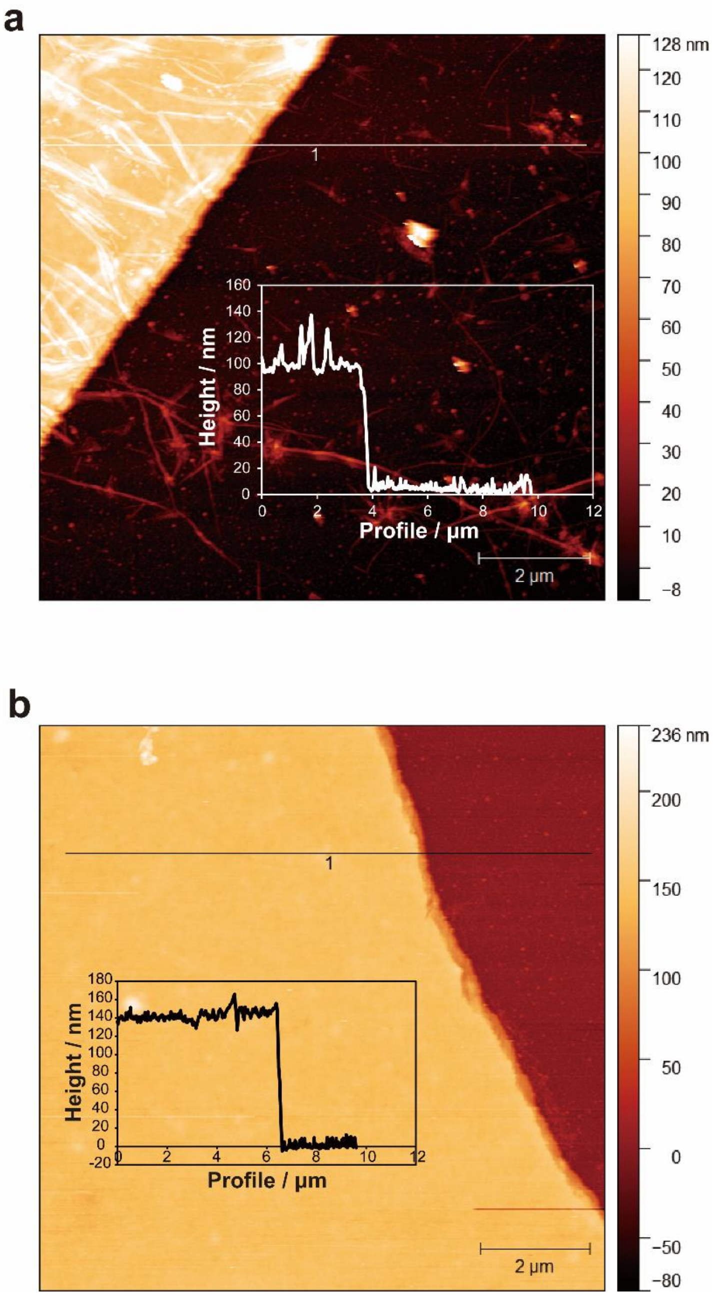

Figure S11. AFM images of (a) **Co-tpy** and (b) **Co/Fe-tpy**.

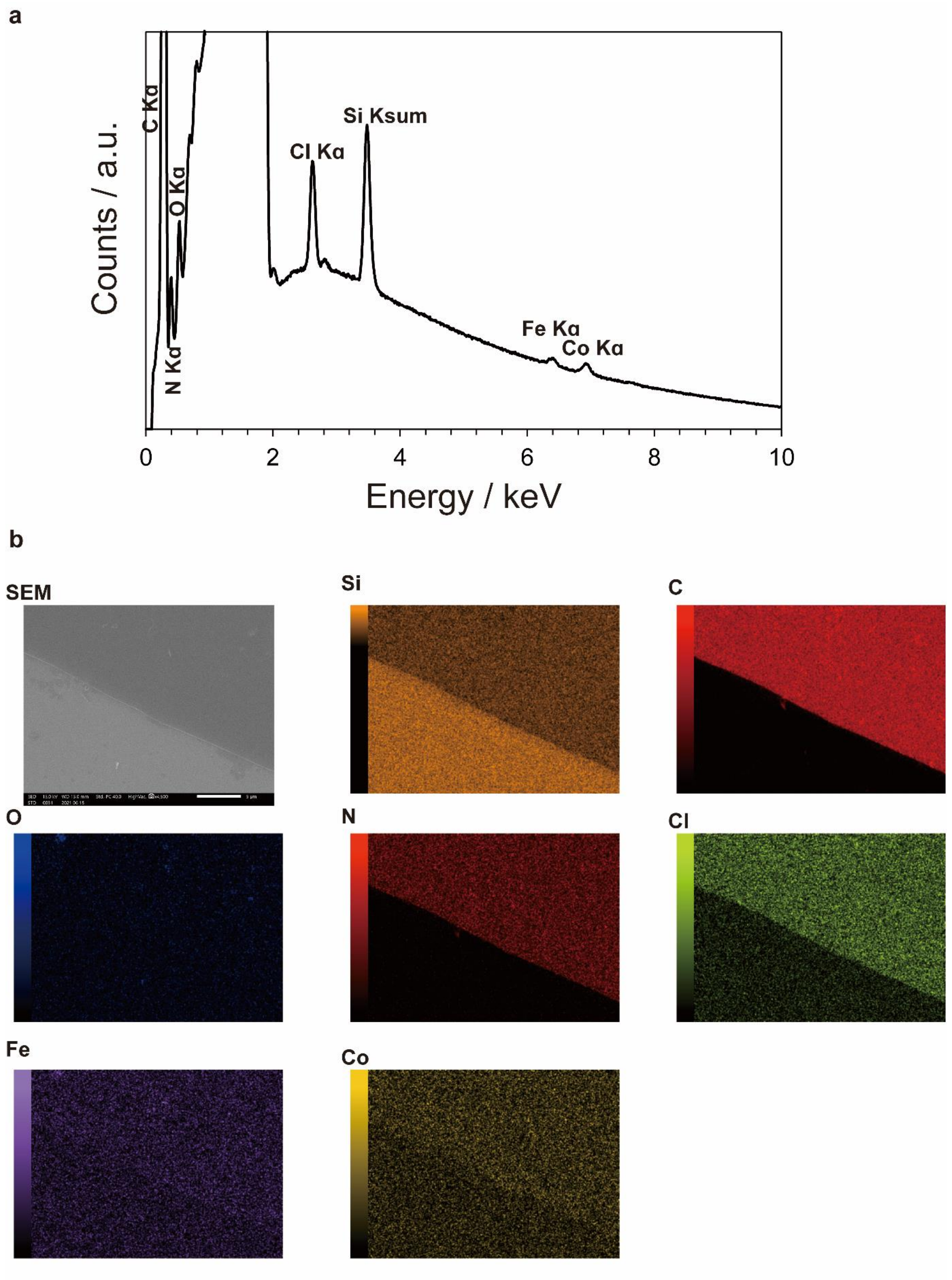

Figure S12. SEM-EDS of **Co/Fe-tpy**. (a) Energy dispersive X-ray spectrum. (b) SEM image and EDS mapping of each element.

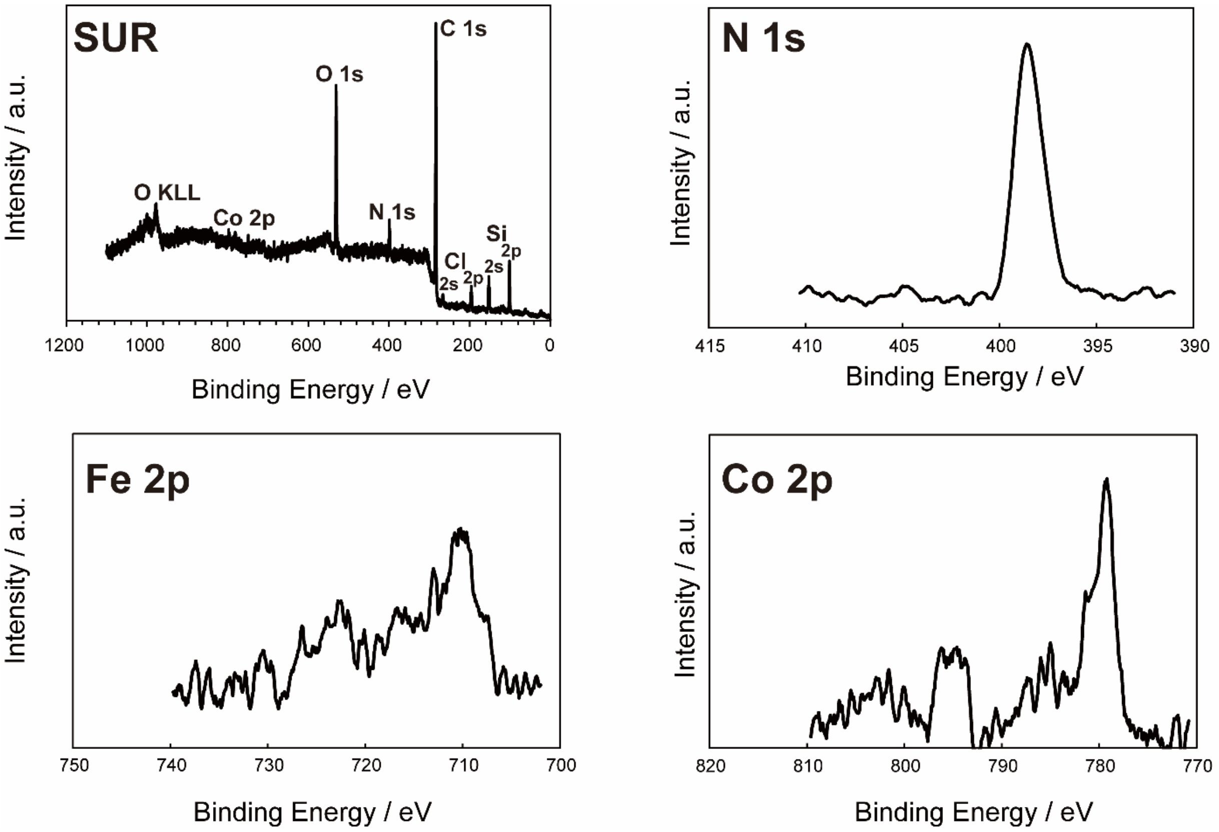

Figure S13. XPS on **Co/Fe-tpy**. Survey scan (top left), Narrow scan of N1s (top right), Fe 2p (bottom left), and Co 2p (bottom right).

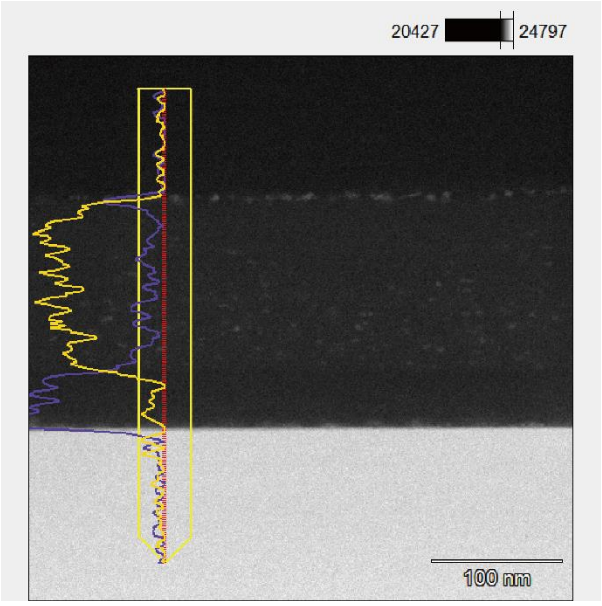

Figure S14. STEM image and EDS line profile across indicated region of **Co/Fe-tpy**. Orange: Co Purple: Fe.

D. Analysis of potential-dependent conductivity of Fe-tpy and Co-tpy

In order to understand the σ-E characteristic of **Fe-tpy** and **Co-tpy** shown in Fig. 3c,d, the electron self-exchange reaction model of Dalton et al.[1] is applied. In this model, the σ-E characteristic of redox polymer films is expressed in eq. 1.

$$\sigma = \theta k_{ex}(nF\delta C_T)^2\chi(1-\chi)/RT \qquad (1)$$

where θ is a geometric factor that varies according to the dimensionality of the polymer film. k_{ex} is the electron self-exchange rate constant. n is the number of electrons. δ is the distance between redox sites at the time of electron transfer. C_T is the concentration of redox sites, and χ is the fraction of oxidized sites.

The σ-E curves of **Fe-tpy** and **Co-tpy** are quite consistent with this model (Fig. 3c,d). For $[Fe(tpy)_2]^{3+/2+}$, δ is estimated at 2.1 nm based on the DFT calculation. When the 155 nm thick **Fe-tpy** with the coverage area of 0.064 cm^{-2} was subjected to cyclic voltammetry, the total charge of the anodic current was 8.2×10^{-5} C corresponding to 8.5×10^{-10} mol of electrons, leading to $C_T = 8.5\times10^{-4}$ mol/cm^{-3}. Using these k_{ex} and C_T values, $k_{ex(CONASH)}$ is estimated at 1.4×10^3 M^{-1} s^{-1}. Our previous study on the redox reaction of the $[Fe(tpy)_2]^{3+/2+}$ couple in dendritic molecular wires constructed with **L1** on the electrode surface, indicated that the electron transfer rate constant between neighbouring $[Fe(tpy)_2]^{3+}$ and $[Fe(tpy)_2]^{2+}$ sites, k_2 is 4.7×10^{12} $cm^2mol^{-1}s^{-1}$,[2] which can be converted to $k_{ex(wire)} = k_2\delta = 9.9 \times 10^3$ $M^{-1}s^{-1}$ using δ = 2.1 nm.

E. Electrochemical analysis of Fe/Co-tpy

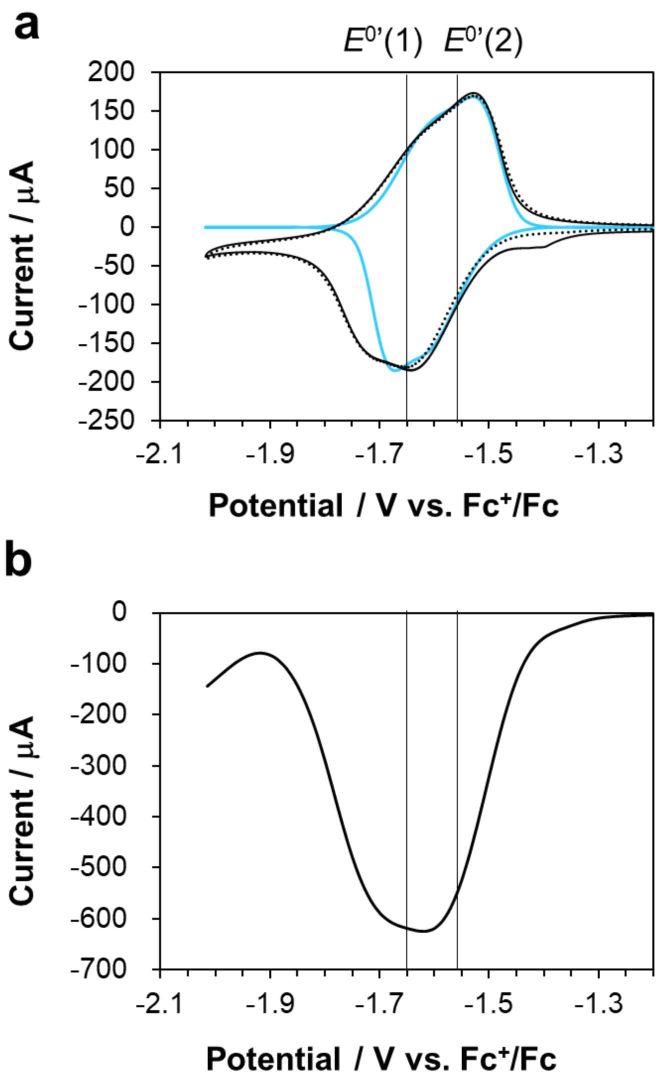

Figure S15. (a) Black lines: Magnified cyclic voltammogram of **Fe/Co-tpy** shown in Figure 3b. Dotted line represents first cycle, while solid line represents second cycle. Light blue line: Simulated voltammogram with E^{0}'(1) = -1.65 V and E^{0}'(2)= -1.56 V. (b) Differential pulse voltammogram of **Fe/Co-tpy**.

F. Electrical conductivity measurement on Fe/Co-tpy and Co/Fe-tpy

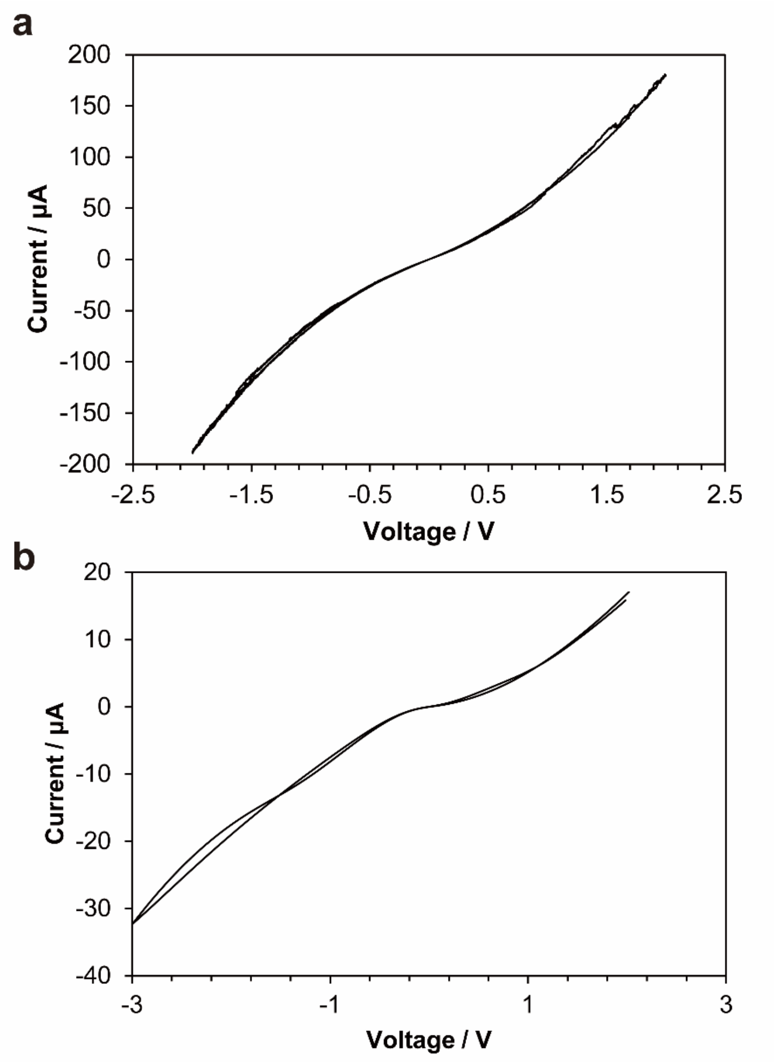

Figure S16. $i-V$ curves of (a) **Fe-tpy** and (b) **Co-tpy**.

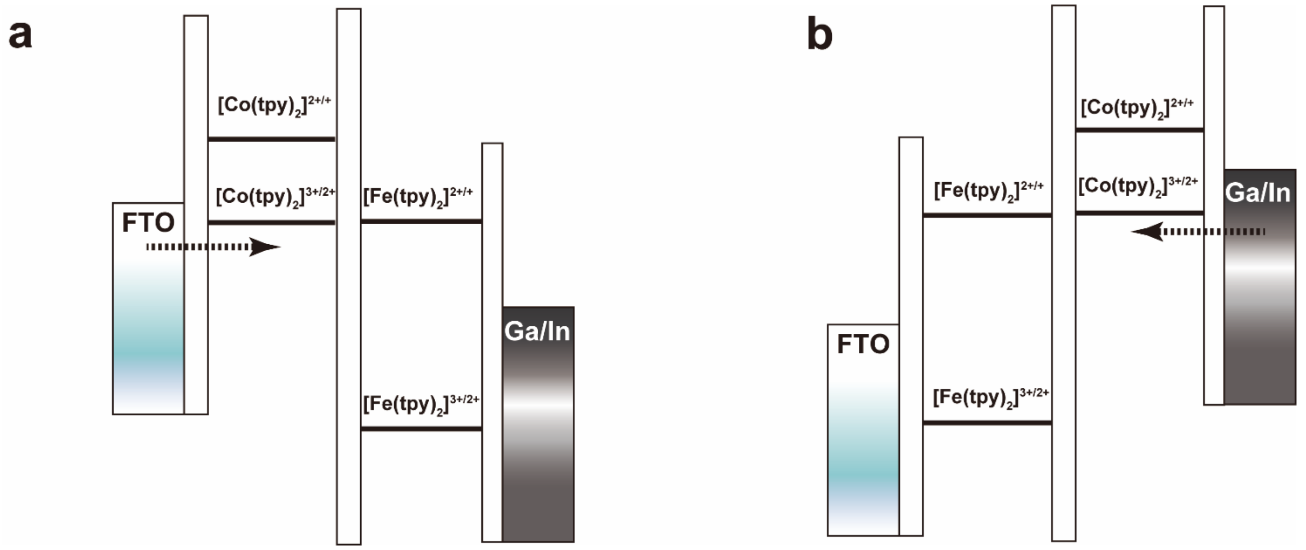

Figure S17. Energy diagram of **Fe/Co-tpy** (a) and **Co/Fe-tpy** (b) applied reverse bias. Dotted arrow represents the path of electron, where slow redox of $[Co(tpy)_2]^{2+/3+}$ prohibit electron to go through.

References

[1] E. F. Dalton, N. A. Surridge, J. C. Jernigan, K. O. Wilbourn, J. S. Facci, R. W. Murray, *Chem. Phys*. **1990**, *141*, 143-157.

[2] Y. Nishimori, K. Kanaizuka, M. Murata, H. Nishihara, *Chem. Asian J.* **2007**, *2*, 367-376.
